# Editorial: Esophageal Stenosis in Esophageal Atresia and Chemical Burn: Update on an Unsolved Problem

**DOI:** 10.3389/fped.2022.1039608

**Published:** 2022-11-01

**Authors:** S. Loff

**Affiliations:** Department of Pediatric Surgery, Olgahospital, Klinikum Stuttgart, Stuttgart, Germany

**Keywords:** children, esophageal stenoses, esophageal atresie, esophageal burn, refractory strictures, therapy

**Editorial on the Research Topic** Esophageal Stenosis in Esophageal Atresia and Chemical Burn: Update on an Unsolved Problem

## Introduction

Esophageal stenoses are frequent problems that occur after esophageal burn or anastomoses in children. It is currently unlikely that this research topic will uncover the solution to this problem. However, our efforts might add a few new views and insights to better understand the problem and to help improve the treatment. Esophageal stenoses not only severely affect the patients and their families due to the multiple interventions and long-term admissions, but also cause considerable costs which need to be cleared by health insurances and the medical community. All are sufficient motives to strive to increase the knowledge about prevention and treatment of this disease.

Five manuscripts have been selected for publication. Three of them deal with the treatment of esophageal stenoses itself, while the other two discuss the prevention of stenoses and long-term consequences of esophageal atresia.

## Prevention

Prevention of injury has been very successfully used in the case of burn and scald injuries. Massive campaigns from patient organizations over the years have led to significantly reduced injuries in developed countries (1). Likewise, prevention of esophageal damage from acid burns or button batteries would be the best way to avoid esophageal stenoses. Indeed, taping the poles from button batteries has been advocated for and experimentally proven to prevent damage (2). Wenyuan et al. investigated the effect of various solutions to mitigate esophageal injury induced by button battery ingestion. This is an experimental study on pig esophageal segments and living piglets. The group found out that irrigation of the esophagus with a mixture of olive oil and honey significantly reduces the discharge of the button battery and thus the damage on the esophagus. Whether or not this procedure will be generally propagated remains to be seen. The benefit of reduced damage has been carefully weighed against the risk of aspiration before anesthesia.

## Therapy

The most common treatment of esophageal stenoses is balloon dilatation (3). Other procedures include bouginage, incision of the stenosis, and stent implantation. Zhou et al. reported the treatment of stenoses in 15 children with dilatation, stenting, and incision after chemical burn of the esophagus or surgical repair of esophageal atresia. Fourteen patients received balloon dilatation as first treatment, which was successful in two patients only. The other patients received additional stenting, endoscopic incision, or both. There were no complications. The procedures were successful in all but four patients who subsequently underwent surgical therapy.

Frequently, adjuncts to dilatation and bouginage are used to increase the effectiveness of dilatation. Cortisone injections and Mitomycin C applications are used, although there is no consensus about many parts of this therapy (4). The meta-analysis and review from Annefleur van Hal et al. is an update of great importance on the question of the safety of intralesional steroid injections in young children and their effectiveness in anastomotic strictures. The authors included 55 studies to address the question of safety of intralesional steroids, which were largely studies about the treatment of hemangiomas. Per patient, 1.67 were performed. Complications were divided into local and systemic. Local complications after intralesional steroid injection occurred in 10% whereas systemic complications were much lower at 0.7%. Systemic complications comprised adrenal insufficiency, Cushingoid syndrome, and growth retardation. The effectiveness of steroid injection in strictures after esophageal atresia reconstruction was assessed in four studies. This meta-analysis suggests that low dose injections of triamcinolone are safe. Furthermore, it is likely that they decrease the number of dilatations necessary for the patient to swallow and thrive.

The esophageal diameter of neonates, children, and adolescents has not been established yet. Consequently, dilatations are carried out using certain arbitrary diameters or, for instance, the “rule of thumb”. Loff et al. investigated the diameters of esophaguses at all ages using esophagograms carried out mainly for exclusion of gastroesophageal reflux or foreign bodies. One hundred and eight patients were selectable in whom the esophagogram showed a bolus in two planes at two different levels. The diameters were plotted against the bodyweight of the patients. The diagram showed a high correlation with the regression line, establishing the median diameter at a given bodyweight. Also, a considerable variation of the diameters at a certain weight was noticed.

## Long-term sequelae

Children with esophageal atresia frequently suffer from long-term sequelae of the esophagus, namely esophageal stenosis and gastroesophageal reflux, but also from associated malformations as well. This makes it important to ensure a lifelong follow-up for these patients, which is stressed by patient advocacy organizations like KEKS (Patienten- und Selbsthilfeorganisation für Kinder und Erwachsene mit kranker Speiseröhre) and EAT (Esophageal Atresia Global support groups) (5). Bisson et al. reported an observation in patients with esophageal atresia during long-term follow-up, which has not been addressed so far. In a cohort of 123 patients with esophageal atresia, kyphosis was found in 25% of patients, thus doubling the frequency of this problem in the general pediatric population. The anomaly was associated with minor orthopedic abnormalities and with the VACTERL-syndrome. For this reason, patients with esophageal atresia should be screened systematically to correct this anomaly early by physiotherapy, self-exercises, or even orthopedic brace-therapy.

This research topic will hopefully add some new grains of knowledge to the complex problem of esophageal atresia and esophageal stenoses. It is the nature of new results to be challenged and verified by more investigations. Thus, further effort is needed to increase safety and improvement of therapy and follow-up of these challenging patients.

## Conflict of interest

The authors declare that the research was conducted in the absence of any commercial or financial relationships that could be construed as a potential conflict of interest.
